# *KMT2C* mutation as a predictor of immunotherapeutic efficacy in colorectal cancer

**DOI:** 10.1038/s41598-024-57519-8

**Published:** 2024-04-09

**Authors:** Chunhua Ni, Xiaohong Wang, Shaoping Liu, Junling Zhang, Zhongguang Luo, Bei Xu

**Affiliations:** 1 Department of Gastrointestinal Surgery, Nanjing Jiangning Hospital of Chinese Medicine, Nanjing, China; 2 Department of General Surgery, Huadong Hospital Affiliated to Fudan University, Shanghai, China; 3 Department of Oncology, Jiangyin People’s Hospital, Jiangyin, China; 4 The Medical Department, 3D Medicines Inc, Shanghai, China; 5 Department of Digestive Disease, Huashan Hospital, Fudan University, Shanghai, 200040 China; 6 Department of Medical Oncology, Cancer Center, Zhongshan Hospital, Fudan University, Shanghai, 200032 China

**Keywords:** Colorectal cancer, Immunotherapy, Tumor mutational burden, Microsatellite instable, Immune cell infiltration, Cancer, Cancer therapy, Tumour biomarkers

## Abstract

Immunotherapy had shown good antitumor activity in a variety of solid tumors, but low benefit in CRC, so there was an urgent need to explore new biomarkers. We evaluated the role of *KMT2C* using publicly available data from the Cancer Genome Atlas (TCGA) and Memorial Sloan Kettering Cancer Center (MSKCC). In addition, further analysis was performed in an internal cohort. Moreover, the mutant profiles of *KMT2C* was analyzed in a large CRC cohort. The relationship between clinical pathologic features and *KMT2C* were analyzed with using the two-sided chi-squared test or the Fisher exact test. Clinicopathologic characteristics associated with overall survival using Cox regression and the Kaplan–Meier method. We found that *KMT2C*-mutated CRC patients in the immunotherapy cohort had significantly improved OS compared with *KMT2C* WT patients (P = 0.013). However, this phenomenon did not exist in non-immunotherapy cohort. Our cohort validated the value of *KMT2C* mutations in predicting better clinical outcomes, including ORR (P < 0.0001) and OS (P = 0.010). Meanwhile, *KMT2C* mutation was associated with higher tumor mutation burden, MSI score, higher levels of immune-associated T cells, neutrophil, and M1-type macrophages. Our study suggested that *KMT2C* mutation might be a potential positive predictor for CRC immunotherapy.

## Introduction

Colorectal cancer (CRC) is a prevalent malignancy globally, ranking among the top three in terms of both incidence and mortality rates^1^. Despite the potential benefits of enhanced colonoscopy screening and the removal of precursor lesions in improving the occurrence and prognosis of CRC, a considerable proportion of patients continue to present with advanced disease. Moreover, the 5-year survival rate for metastatic CRC remains below 15%^2^. The advent of immunotherapy marked a significant advancement in the field of cancer treatment. Nevertheless, the effectiveness of immune checkpoint inhibitors (ICIs) in clinical settings exhibits considerable variability. Presently, numerous ICIs, including pembrolizumab, have demonstrated encouraging efficacy and have received approval from the United States Food and Drug Administration for the management of advanced CRC tumors characterized by high levels of microsatellite high instability or mismatch repair deficiency (MSI-H/dMMR)^3^. Despite MSI-H/dMMR being the established biomarker for immunotherapy in CRC, only 4–7% CRC patients were MSI-H/dMMR^4^, and some of these patients may still experience resistance to immunotherapy^5^. The effectiveness of other potential biomarkers, such as tumor mutation burden (TMB) and PD-L1 expression, in predicting response to immunotherapy in CRC remains uncertain^6^. Consequently, further investigation is required to identify additional predictive biomarkers.

There is a growing body of evidence indicating the significant role of epigenetic factors in anti-tumor mechanisms^7^. The KMT2 family genes, which play a crucial role in epigenetic regulation. Specifically, *KMT2C*, a member of the KMT2 family genes, also referred to as myeloid/lymphoid or mixed lineage leukemia protein 3, has been identified as highly expressed in various cancers such as acute myeloid leukemia, low-grade glioma, and thymoma^8^. Furthermore, research has demonstrated that elevated levels of KMT2C expression are independently correlated with reduced overall survival (OS) in lung adenocarcinoma^9^. Additionally, *KMT2C* mutations in diffuse gastric adenocarcinoma have been found to promote epithelial-to-mesenchymal transition and are associated with a poorer OS outcome^10^. Significantly, the enhancement of PD-L1 transcription by *KMT2C* and its influence on anti-tumor immunity imply that *KMT2C* mutations hold potential as a biomarker for tumor immunotherapy^11^. However, the relationship between the *KMT2C* gene and immunotherapy for CRC remains unexplored.

This study aims to examine the relationship between *KMT2C* mutations and survival in CRC using two publicly available datasets and an internal CRC dataset. The study characterizes the mutation feature of *KMT2C* in CRC and explores its predictive value for CRC immunotherapy efficacy. Furthermore, a larger sample cohort (3Dcohort) is utilized to investigate the mutation frequency and profile of *KMT2C* in Chinese patients with CRC. Additionally, the study analyzes several immune-related characteristics, such as MSI status and TMB, to identify their correlation with *KMT2C* mutations in CRC. We aimed to provide a comprehensive outline of *KMT2C* mutations and propose a potential predictor for immunotherapeutic efficacy in CRC.

## Results

### *KMT2C* mutations in tumor

**Figure 1:**
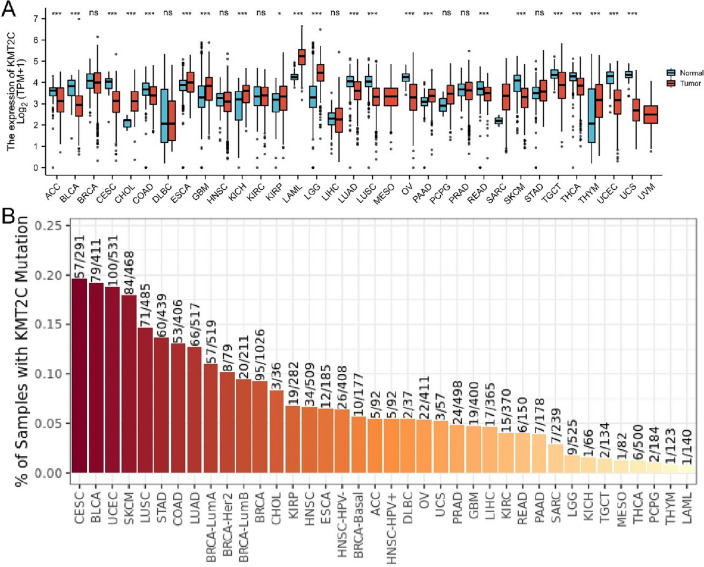
*KMT2C* profile in different types of human cancers. (**A**) *KMT2C* expression levels in different tumor types from UCSC XENA database. (**B**) *KMT2C* mutation proportion in different tumor types from TCGA database were determined by TIMER. (ns P > 0.05, *P < 0.05, **P < 0.01, ***P < 0.001).

In order to investigate the involvement of the *KMT2C* gene in tumorigenesis, we conducted an analysis utilizing data from XENA, TCGA, and GTEx databases to assess the transcriptional activity of *KMT2C* mRNA in both tumor and normal tissue. Our results indicate that, with the exception of breast cancer, lymphoma, head and neck cancer, kidney cancer, pheochromocytoma and paraganglioma, prostate cancer and stomach cancer, the expression level of *KMT2C* mRNA in tumor tissue exhibited significant alterations compared to that in normal tissue (Fig. 1A). Additionally, *KMT2C* mutations were observed at a frequency exceeding 10% in 10 different types of cancer, including CRC (Fig. 1B). These findings provide compelling evidence suggesting that *KMT2C* may play a crucial role in the pathogenesis of cancer.

### Association between *KMT2C* mutations and benefit to ICIs in patients with CRC

**Figure 2:**
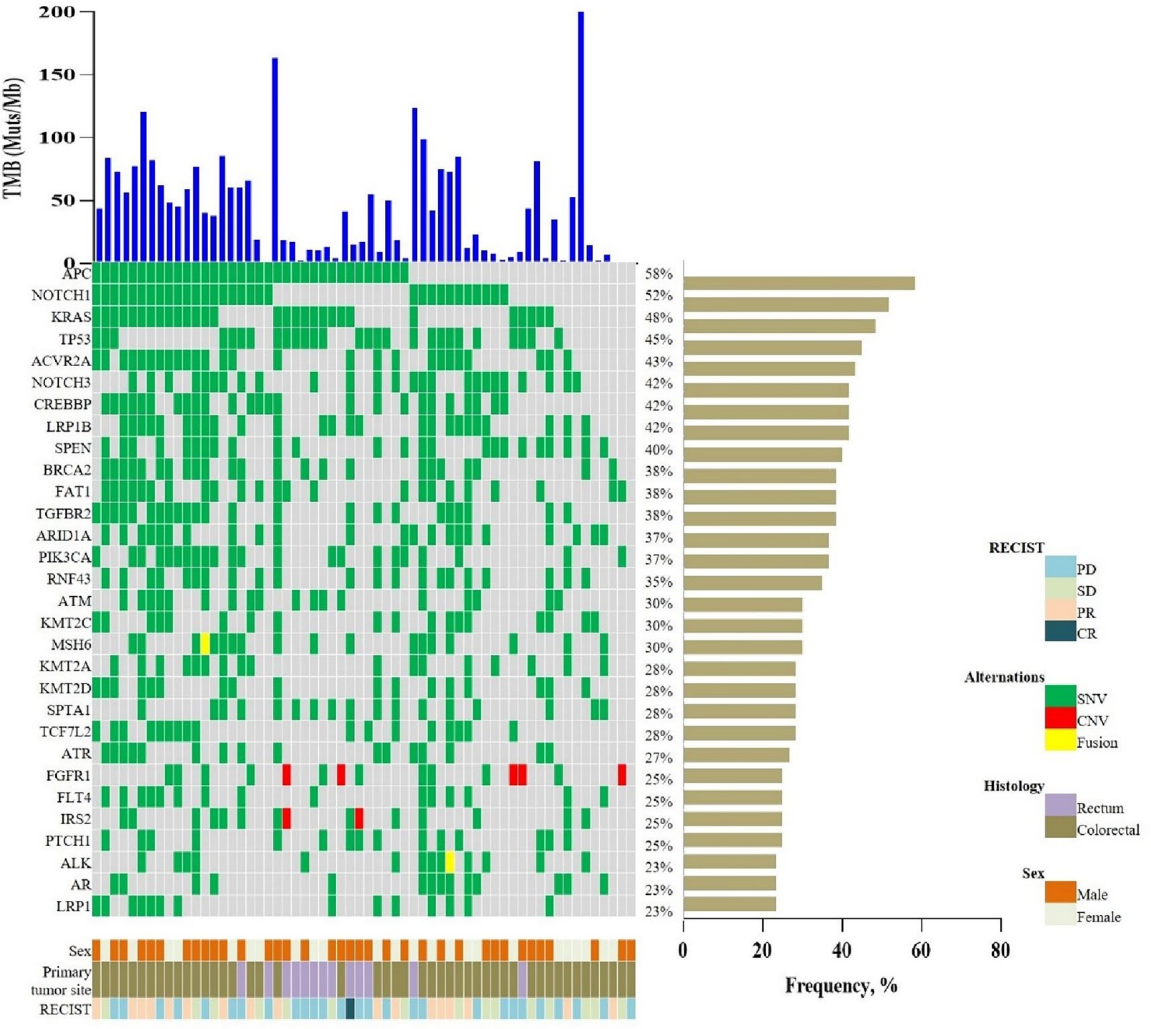
The genomic characteristics of CRC patients in the internal cohort.

The baseline characteristics of CRC patients in both the MSKCC cohort and the internal cohort were comprehensively summarized in Table 1. Among the MSKCC cohort, a total of 20 patients (18.2%) were found to carry the *KMT2C* mutation (KMT2C-MUT), while the remaining 90 patients were classified as *KMT2C* wild-type (KMT2C-WT). Within the internal cohort, 18 patients (30%) were identified as KMT2C-MUT, with the K2797Rfs*26 (6/18, 33%) mutation site being the most prevalent among the *KMT2C* mutations. Among the 60 patients in the internal cohort, 36 were male and 9 patients were over the age of 65. Notably, the KMT2C-MUT group exhibited a higher proportion of MSI-H cases in both cohorts (P < 0.0001, P = 0.041, respectively). However, no significant differences in age sex, primary site and tumor differentiation were observed between the KMT2C-MUT and KMT2C-WT groups. The genomic mutation landscape of internal cohort were displayed in Fig. 2. Mutations in *APC*, *NOTCH1*, *KRAS*, *TP53* and *ACVR2A* were more frequently observed.

**Table 1:** Baseline characteristics in the MSKCC and internal cohorts.

Characteristics	MSKCC cohort			Internal cohort		
	KMT2C-MUT (n = 20)	KMT2-WT (n = 90)	P value	KMT2C-MUT (n = 18)	KMT2C-WT (n = 42)	P value
Age, n (%)						
≥ 65	6 (30)	21 (23)	0.570	2 (11)	7 (17)	0.710
< 65	14 (70)	69 (77)		16 (89)	35 (83)	
Sex, n (%)						
Male	14 (70)	48 (53)	0.217	9 (50)	27 (64)	0.391
Female	6 (30)	42 (47)		9 (50)	15 (36)	
Primary tumor site, n (%)						
Rectum	2 (10)	16 (18)	0.519	0 (0)	13 (31)	0.011
Colorectal	18 (90)	74 (82)		18 (100)	29 (69)	
Tumor differentiation, n (%)						
High + medium	–	–	–	17 (94)	31 (74)	0.086
Low	–	–		1 (6)	11 (26)	
MSI status, n (%)						
MSI-H	15 (75)	20 (22)	< 0.0001	15 (83)	22 (52)	0.041
MSS	5 (25)	70 (78)		3 (17)	20 (48)

**Figure 3:**
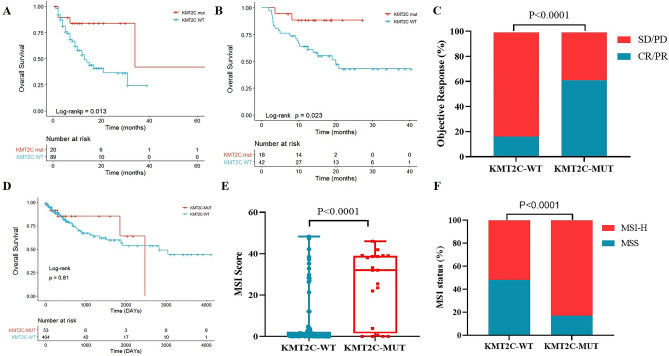
Association of *KMT2C* status and clinical outcomes. Kaplan–Meier curves of overall survival according to *KMT2C* mutation status in the MSKCC cohort (**A**) and internal cohort (**B**). (**C**) Histogram depicting proportions of patients achieved objective response (CR/PR) and stable disease (SD) in *KMT2C*-MUT and *KMT2C*-WT groups. (**D**) Kaplan–Meier curves of overall survival according to *KMT2C* mutation status in the TCGA cohort. Comparison of MSI score between the *KMT2C*-MUT and *KMT2C*-WT tumors in the MSKCC cohort (**E**) and internal cohort (**F**).

To investigate the correlation between *KMT2C* mutations and the effectiveness of ICIs in patients diagnosed with CRC, an analysis was initially conducted in the MSKCC cohort. The results depicted in Fig. 3A demonstrate a statistically significant improvement in OS for patients with KMT2C-MUT compared to those with KMT2C-WT (median OS: 13.0 vs 34.0 months, HR = 0.31, 95% CI 0.15–0.61, P = 0.013). This finding was further validated within our internal cohort, where *KMT2C* mutations were associated with better OS (Fig. 3B, HR = 0.17, 95% CI 0.05–0.57, P = 0.023) and a higher objective response rate (ORR, 61% vs 17%, P < 0.0001, Fig. 3C). However, this phenomenon was not observed in the non-immunotherapy cohort (TCGA cohort, Fig. 3D). In addition, we combined data from three cohorts (MSKCC cohort, TCGA cohort and internal cohort) to analyze whether there was a difference in prognosis between patients with *KTM2C* mutations and wild-type patients. There was no significant difference in overall survival between KMT2C-mut patients and wild-type patients (P = 0.700, Supplement Fig. S1). Also included in other solid tumors, *KMT2C* did not predict the prognosis of immunotherapy (Supplement Fig. S2). Furthermore, it was observed that patients with *KMT2C* mutations exhibited higher MSI scores in both immunotherapy cohorts (both P < 0.0001; Fig. 3E,F).

In univariate analysis, *KMT2C* gene, MSI status, and sex were independent prognostic factors in the MSKCC cohort. This result is further verified in the internal cohort. *KTM2C* mutation, tumor site, and tumor differentiation were significantly associated with OS in the internal cohort (Table 2). Thus, our results suggested that *KMT2C* gene might be a potential predictive biomarker for CRC patients undergoing ICIs therapy.

**Table 2:** Hazard ratio (HR) of clinical and genomic variables on overall survival via univariate and multivariate analysis in the MSKCC cohort and internal cohort.

Parameter	Univariate analysis			Multivariate analysis		
	HR	95% CI	P	HR	95% CI	P
**MSKCC cohort**						
Sex						
Male vs female	0.534	0.295–0.967	0.038	0.498	0.272–0.911	0.024
Age						
≥ 65 vs < 65	0.457	0.203–1.025	0.057			
*KMT2C* gene						
MUT vs WT	0.288	0.102–0.817	0.019	0.534	0.170–1.681	0.284
Tumor location						
Rectum vs colorectal	0.662	0.260–1.681	0.385			
MSI status						
MSI-H vs MSS	0.313	0.138–0.707	0.005	0.361	0.147–0.889	0.027
**Internal cohort**						
Sex						
Male vs female	1.158	0.489–2.743	0.738			
Age						
≥ 65 vs < 65	2.152	0.796–5.821	0.131			
Tumor differentiation						
High + medium vs Low	0.010	0.001–0.081	0.000	0.011	0.001–0.095	0.000
*KMT2C* gene						
MUT vs WT	0.216	0.050–0.924	0.039	0.271	0.058–1.272	0.098
Tumor location						
Rectum vs colorectal	2.667	1.152–6.172	0.022	1.444	0.470–4.439	0.522
MSI status						
MSI-H vs MSS	0.594	0.260–1.355	0.215	1.400	0.456–4.298	0.557

Since the US Food and Drug Administration (FDA) approved Pembrolizumab for the treatment of advanced solid tumors with dMMR/MSI-H, MSI has jumped to the forefront of cancer molecular diagnosis and is an important predictor of bowel cancer^12^. Therefore, by using a two-step classification strategy, patients were divided into four subgroups: KMT2C-wt and MSS, KMT2C-wt and MSI-H, KMT2C-MUT and MSS, and KMT2C-MUT and MSI-H. We found that patients with *KMT2C* mutations were more likely to be MSI-H (Supplement Fig. S3A,B), but the two were not the same. Patients with *KMT2C* mutations combined with MSI-H had the best prognosis (Supplement Fig. S3C). This phenomenon is also repeated in internal queues (Supplement Fig. S3D). Accordingly, KMT2C-MUT and MSI-H subgroup was the most sensitive to ICI, followed by KMT2C-MUT and MSS subgroup and KMT2C-wt subgroup (66.7% vs 33.3% vs 17.1%, P < 0.0017, Supplement Fig. S3E).

### *KMT2C* mutation was indicative of an immune-hot status

**Figure 4:**
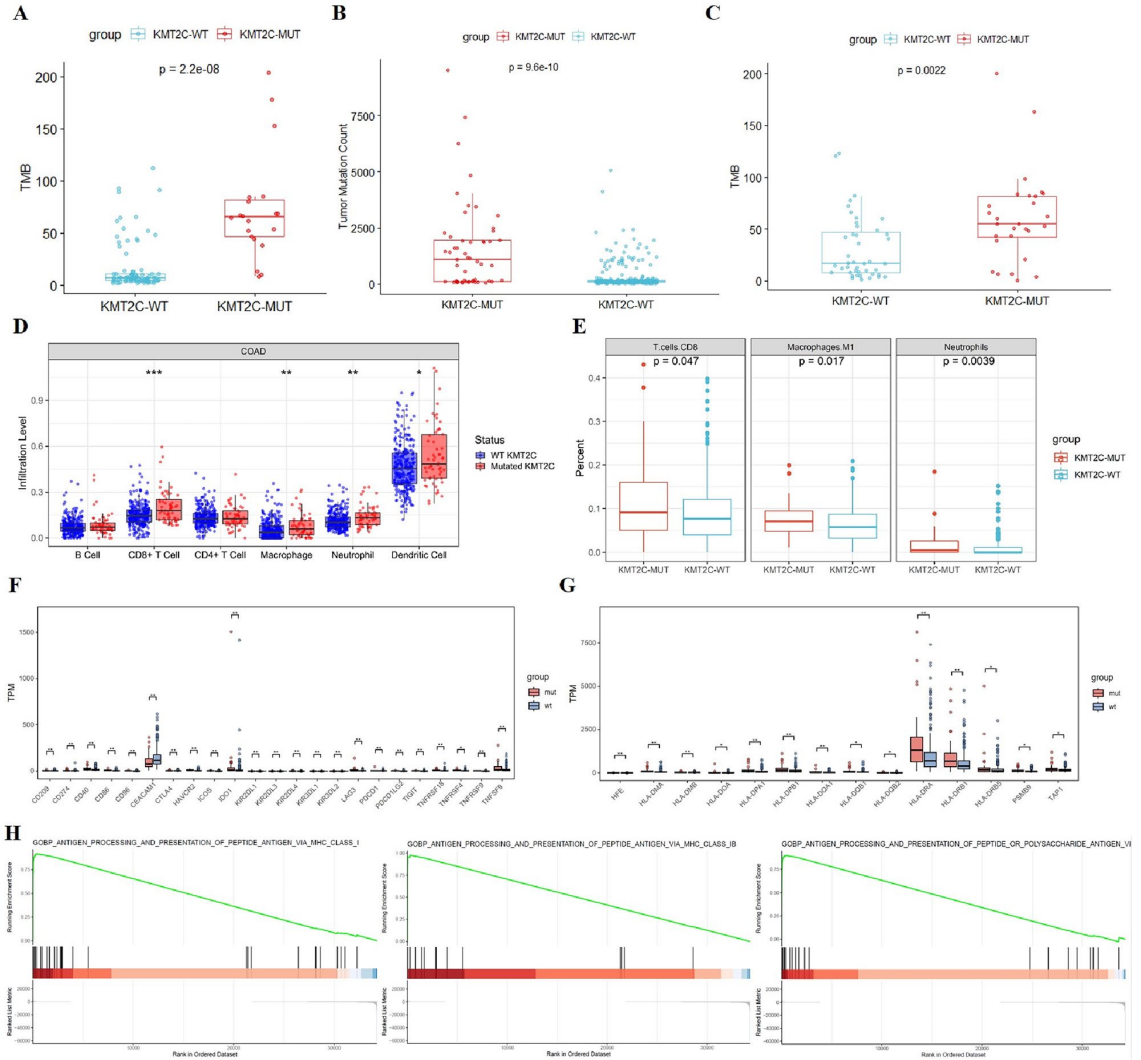
The relationship between *KMT2C* mutations and immune infiltration level. (**A**–**C**) Comparison of TMB between KMT2C3-MUT and KMT2C-WT patients in MSKCC cohort, TCGA cohort and internal cohort, respectively. Box plots are generated from TIMER (**D**) and cibersort (**E**). (**F**) Boxplot of differentially expressed immune-related genes in KMT2C-MUT and KMT2C-WT groups. (**G**) Boxplot of differentially expressed HLA family genes in KMT2C-MUT and KMT2C-WT groups. (**H**) GSEA reveals prominent enrichment of signatures related to antigen processing and presentation in CRC patients with KMT2C mutation. *P < 0.05, **P < 0.01, ***P < 0.001. *TMB* tumor mutational burden.

In order to gain a deeper understanding of the underlying mechanism linking *KMT2C* mutation and improved clinical outcomes in CRC patients who underwent ICIs treatment, an investigation was conducted to assess the impact of *KMT2C* mutation on TMB or immune signatures. The findings presented in Fig. 4A–C indicate a positive correlation between *KMT2C* mutation and elevated TMB all in MSKCC cohort, TCGA cohort and internal cohort (all *P* < 0.05). Utilizing the TIMER tool, it was observed that KMT2C-MUT tumors exhibited higher levels of CD8+ T cells, macrophages, neutrophils, and dendritic cells (DC) (Fig. 4D). Similarly, the cibersort analysis revealed an increased infiltration of CD8+ T cells, neutrophils, and M1 macrophages in KMT2C-MUT tumors (Fig. 4E). These results collectively suggest that *KMT2C* mutation is associated with a hot immune status and may play a role in enhancing the immune response.

Furthermore, PD-L1 (CD274) and CTLA4 were significantly up-regulated in KMT2C-MUT tumors (Fig. 4F). I The HLA family was more prevalent in the KMT2C-MUT tumors (Fig. 4G). In addition, KMT2C-MUT tumors had higher expression of MHC I- and II-associated antigen-presenting molecules than KMT2C-WT tumors (Fig. 4H). These results suggest that KMT2C-MUT is associated with a hot immune status and enhances the efficacy of ICBs.

### Mutational profiles of CRC in 3Dcohort

**Figure 5:**
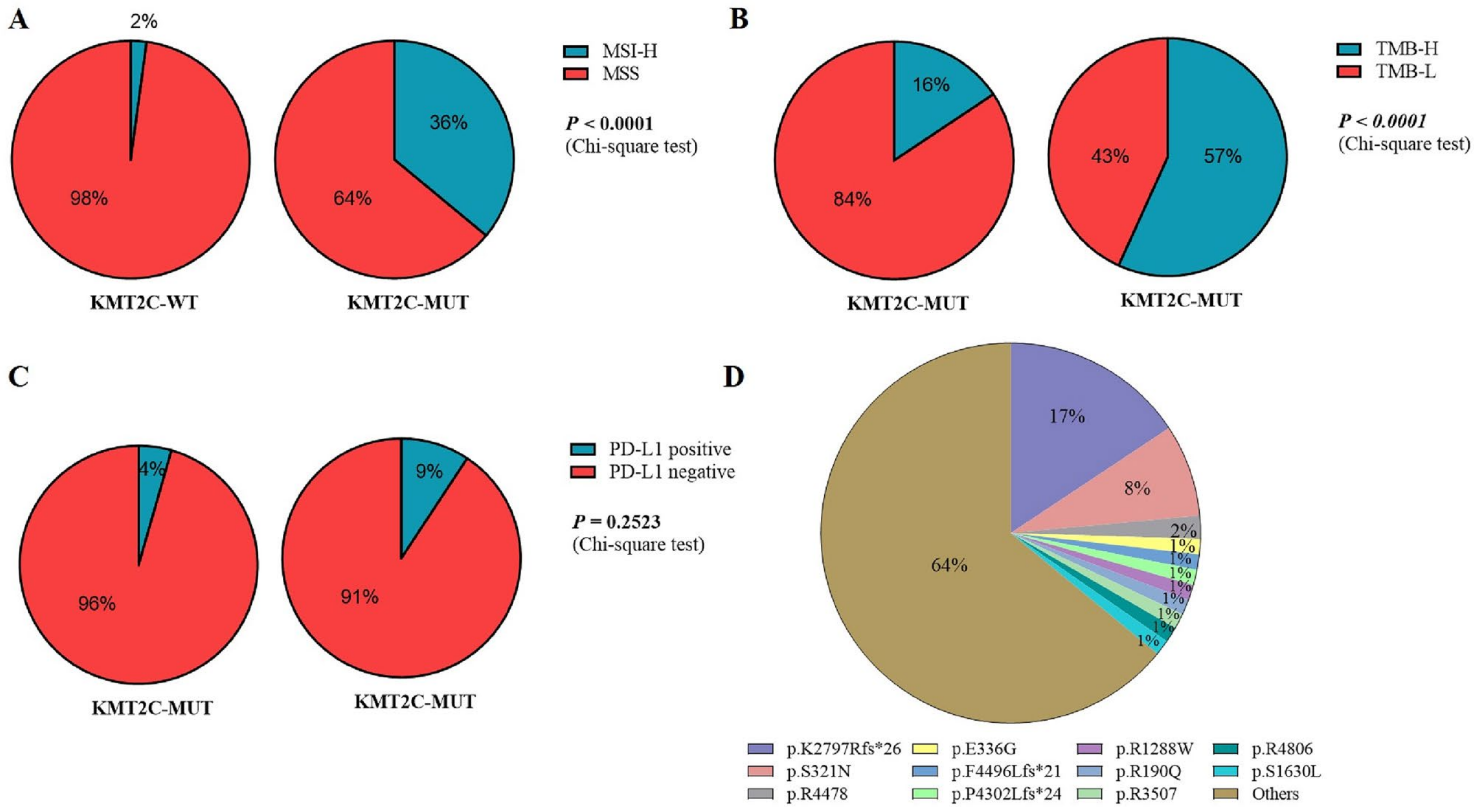
The relationship between *KMT2C* mutations and microsatellite status (**A**) or tumor mutational burden (**B**) or PD-L1 status (**C**) in the 3Dcohort. (**D**) Pie charts of *KMT2C* mutations sites.

In order to investigate the mutational profile of *KMT2C* in a large population of CRC patients, a comprehensive analysis was conducted on 905 individuals who had undergone tissue NGS, including 316 rectal and 589 colorectal cancer patients (Table 3). The findings revealed that the occurrence of *KMT2C* mutations in 111 CRC patients was lower compared to the MSKCC and internal groups (MSKCC group: 12.2% vs 18.2%, P = 0.0962; Internal cohort: 12.2% vs 30.0%, P = 0.0005), but similar to the TCGA cohort (12.2% vs 11.6%, P = 0.7915). This discrepancy may be attributed to the specific characteristics of the cohort. Furthermore, the *KMT2C* mutant group exhibited an enrichment of patients with MSI-H and TMB-H (P < 0.0001, Fig. 5A,B). However, no significant correlation was observed between PD-L1 expression and *KMT2C* mutations (P = 0.2523, Fig. 5C). Notably, the most frequently mutated site in *KMT2C* was identified as K2797Rfs*26, followed by *S321N* and *R4478*, and 64% of the sites was detected only once (Fig. 5D).

**Table 3:** Baseline characteristics of the 3Dcohort.

Characteristics	Total (N = 905)	KMT2C-MUT (n = 111)	KMT2C-WT (n = 794)
Age, n (%)			
≥ 65	273 (30)	35 (32)	238 (30)
< 65	632 (70)	76 (68)	556 (70)
Sex, n (%)			
Male	569 (63)	65 (59)	504 (63)
Female	336 (37)	46 (41)	290 (37)
Primary tumor site, n (%)			
Rectum	316 (35)	26 (23)	290 (37)
Colorectal	589 (65)	85 (77)	504 (63)

## Discussion

In this study, the presence of *KMT2C* mutations was firstly identified as a favorable factor for improved clinical outcomes in CRC patients treated with immune checkpoint inhibitors (ICIs). Conversely, patients receiving standard care did not experience the same clinical benefits in terms of OS. Furthermore, univariate analysis confirmed that *KMT2C* mutation is an independent positive predictor. Exploratory analyses indicated that higher tumor mutational burden (TMB) and MSI scores may serve as potential mechanisms for predicting the value of *KMT2C* mutations in CRC. Utilizing the CIBERSORT, we observed a higher abundance of CD8+ T cells, macrophages, neutrophils, and DCs in *KMT2C*-mutated tumors. These findings suggest that *KMT2C* mutations may play a significant positive predictor role for CRC patients treated with ICIs.

In recent years, ICIs have demonstrated significant efficacy in the management of various cancer types. However, the identification of patients who are likely to benefit from immunotherapy remains a challenging clinical issue due to the variability in clinical responses^13^. Although specific mutations (*PTPRD/PTPRT*, *KDR*, *KEAP1/NFE2L2* and so on) have been implicated in influencing the response to immunotherapy, these genetic alterations lack robust clinical evidence and the frequency of mutations is low^14–16^. Our study proposes that the *KMT2C* gene may serve as a potential biomarker for ICIs in CRC. We have established a correlation between the status of the *KMT2C* gene and the response to immunotherapy. Importantly, our findings were reaffirmed in the internal cohort. Meanwhile, *KMT2C* mutations were not uncommon in colon cancer, at about 8.4%, meaning that *KMT2C* genes would cover more patients than other rare mutations.

*KMT2C*, a prominent epigenetic regulator, exerts a crucial function in transcriptional regulation by inducing relaxation of chromatin structure through its catalytic involvement in the monomethylation of histone H3 lysine K4^17^. Numerous large-scale sequencing investigations have identified *KMT2C* as one of the most frequently mutated genes across diverse cancer types^18^. Notably, mutations in the *KMT2C* gene have been demonstrated to facilitate the transition from epithelial to mesenchymal states in diffuse gastric adenocarcinoma (GAC)^10^. Additionally, *KMT2C* has been implicated in the maintenance of genomic stability, as mutations in this gene lead to a substantial increase in TMB across various cancer types^19^. In this study, a consistent positive correlation was observed between *KMT2C* mutations and elevated tumor mutational burden (TMB) in CRC patients, potentially resulting in heightened levels of neoantigen load (NAL) levels. It has been demonstrated that increased NAL levels significantly augment the capacity of cytotoxic T cells and macrophages to identify cancer cells and elicit an immune response for the eradication of cancer cells, thereby closely aligning with improved clinical outcomes^20^. This finding serves as one of the rationales supporting the association between *KMT2C* mutations and favorable clinical outcomes in the context of immune checkpoint inhibitors (ICIs).

To the best of our knowledge, this study represents the first exploration of the association between *KMT2C* mutations and ICIs in CRC. Notwithstanding, our study is subject to certain limitations, including the inherent constraints of a retrospective design. The analysis was conducted on a general public cohort of cancers that received WES or multi-gene panel sequencing, which might result in selection bias. Nevertheless, our internal cohort was included as a robust complement. Regrettably, the limited number of patients precluded further investigation into the relationship between *KMT2C* mutations and the efficacy of ICIs, but the overall efficacy of *KMT2C* mutations is satisfactory. Of course, above results should be confirmed in large cohorts. In addition, only bioinformatics analysis has been used to investigate the potential mechanism by which *KMT2C* mutations enhance the efficacy of immunotherapy. Our conclusion was only that *KMT2C* mutation was related to TIICs, but the specific mechanism of *KMT2C*-MUT induced immune changes was worthy of further experimental research.

Taken together, our results suggest that in CRC patients receiving ICIs, *KMT2C* mutations were associated with better ORR and OS through increased TMB and immune-related genetic signatures. *KMT2C* mutations might be an important part of the immunogenetic landscape and should be integrated into the predictive biomarker panel for ICIs therapy. Further exploration of molecular mechanism and prospective clinical trials are warranted.

## Materials and methods

### Clinical cohort and public cohorts

In this study, two independent public cohorts were used, including the Cancer Genome Atlas (TCGA) and Memorial Sloan Kettering Cancer Center (MSKCC). The genomic, survival and mRNA data were downloaded, respectively. Concurrently, we conducted a retrospective analysis of patients diagnosed with advanced CRC who received PD-1/PD-L1 inhibitors at our institution, utilizing them as an internal cohort for our study. Prior to immunotherapy, all patients underwent molecular testing. Relevant clinical characteristics, including age, sex, histological type, tumor site, treatment response, survival, and gene variation, were extracted. In addition, a total of 1314 patients with CRC were included in the 3Dcohort. To ensure accuracy and reliability, all patients underwent next-generation sequencing (NGS) using Illumina HiSeq sequencers, with 150 cancer gene plates, in CAP and CLIA-certified laboratories (3D Medicines Inc., Shanghai).

### DNA extraction, library preparation and DNA sequencing

The previous study outlined the experimental techniques employed for NGS, including DNA extraction, library preparation, and DNA sequencing^21^. Hematoxylin and eosin staining was used to assess the tumor cell content in FFPE tissue sections. Only samples with a tumor content equal to or greater than 20% were considered for further analysis. The plasma was separated from the blood by centrifuging it in a Streck tube at 1600*g* for 20 min at room temperature. Subsequently, gDNA was extracted from both tumor FFPE tissue and white blood cells using the DNeasy tissue or blood kit, following a standardized protocol. The libraries were then prepared using the KAPA Hyper Prep Kit (KAPA Biosystems) following the manufacturer’s protocol. The concentration and size distribution of each library were determined using a Qubit 3.0 fluorometer (Thermo Fisher Scientific) and a LabChip GX Touch HT Analyzer (PerkinElmer) respectively. Germline mutations were excluded. All single nucleotide variations (SNVs) and indels in the coding region of *KMT2C* gene, including missense, silent, stop gain, stop loss, in-frame and frameshift mutations, were considered. TMB was defined as the number of nonsynonymous somatic SNVs and indels in examined coding regions, with driver mutations excluded.

### Immune cell infiltration

The infiltration level of immune cells in CRC was analyzed using the TIMER database through gene mutation analysis (https://cistrome.shinyapps.io/timer/)^22^. Specifically, the gene name *KMT2C* within the mutation module was utilized to establish a correlation between *KMT2C* mutation and the extent of immune cell infiltration. The "CIBERSORT" (R package) was employed to determine the proportions of 22 tumor immune infiltrating cell types (TIICs) in each sample^23^. By analyzing the relative expression levels of genes in a single tissue sample, CIBERSORT predicted the proportions of the 22 TIICs based on their gene expression profiles (GEPs). The normalized GEPs of CRC were then converted into the proportions of the 22 TIICs. The relative expression of 22 TIICs in each sample was determined.

### Statistical analysis

The primary outcome was OS, which was calculated from the date of first immunotherapy until death from any cause. Hazard’s ratio (HR) was determined through cox proportional hazards regression model. Continuous variables were analyzed using Student’s *t*-tests or *U*-tests. Categorical variables were analyzed using Chi-squared tests or Fisher's exact tests. Prognostic analyses were performed using Kaplan–Meier survival analysis and Cox univariate and multivariate analyses. Variables with a P-value < 0.10 in the univariable regression were included in the multivariable analyses. Data analyses were performed using SPSS 20.0 (SPSS Inc., Chicago, IL). P < 0.05 were considered statistically significant.

### Reporting summary

Further information on research design is available in the Nature Portfolio Reporting Summary linked to this article.

### Supplementary Information

Supplementary Figures.

## Data Availability

The datasets supporting the conclusions of this article are available in the Cancer Genome Atlas (https://portal.gdc.cancer.gov, COAD and READ category), cBioPortal database (https://www.cbioportal.org), and TIMER database (https://cistrome.shinyapps.io/timer/).
