# Supplementary material for: KMT2C mutation as a predictor of immunotherapeutic efficacy in colorectal cancer
Source: Sci Rep. 2024 Apr 9;14:8284. doi: 10.1038/s41598-024-57519-8 (PMC11004165; doi:10.1038/s41598-024-57519-8)
Supplement: Supplementary file 1 — Supplementary Figures. [file 41598_2024_57519_MOESM1_ESM.docx]

***KMT2C* mutation as a predictor of immunotherapeutic efficacy in colorectal cancer**

**Supplement Figure S1.** Kaplan-Meier curves of overall survival according to *KMT2C* mutation and clinical outcomes regardless of treatment.


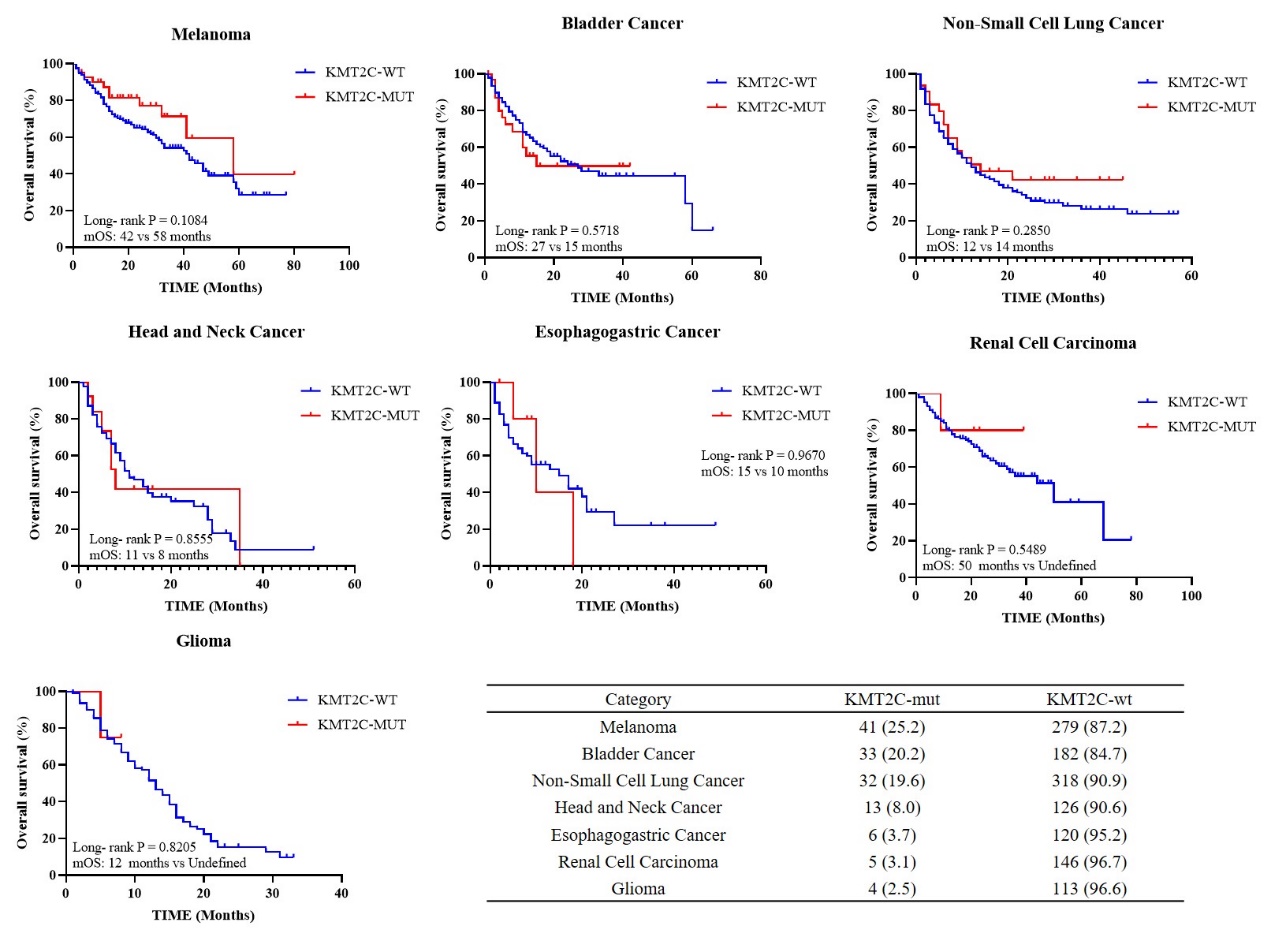


**Supplement Figure S2.** Association of *KMT2C* status and clinical outcomes in others solid cancer.


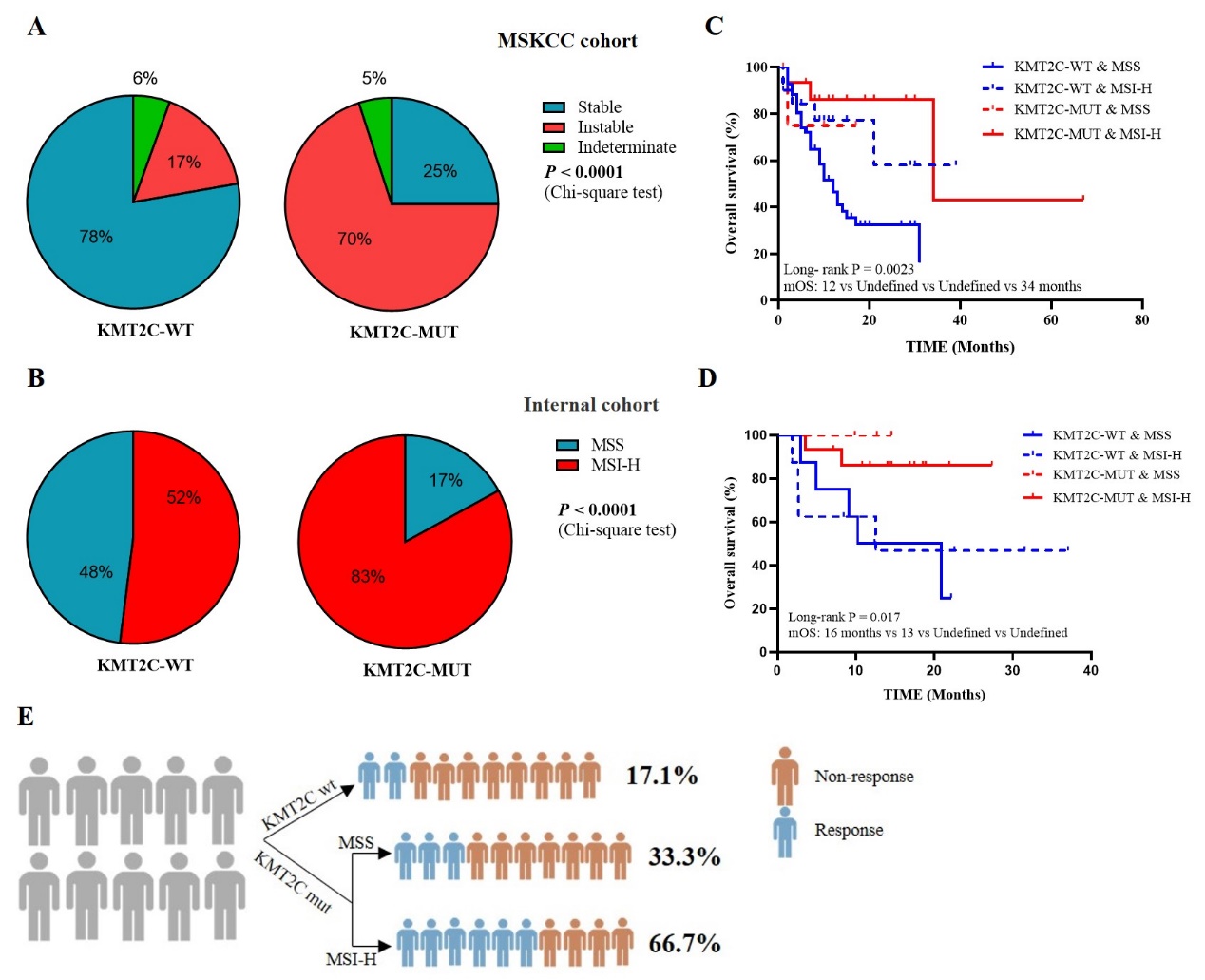


**Supplement Figure S3. The predictive value of MSI in CRC with *KMT2C* mutation.** The percentage of MSI patients in KMT2C-mut and KMT2C-wt patients in the MSKCC cohort (A) and internal cohort (B). Kaplan-Meier curves comparing OS in the MSKCC cohort (C) and internal cohort (D). (E) Through a step-by-step filtering using MSI and KMT2C, patients with CRC are stratified into three subgroups, KMT2C-wt, KMT2C-mut & MSS, and KMT2C-mut &MSI-H, with significantly different rates of response to ICIs.
